# Pregnancy outcomes and the dose-related effects of acupuncture therapies in women undergoing in vitro fertilization: A protocol for systematic review and meta-analysis

**DOI:** 10.1097/MD.0000000000021815

**Published:** 2020-08-28

**Authors:** Zihao Zou, Qianhua Zheng, Xin Wen, Zuoqin Yang, Tinghui Hou, Xinyu Hao, Siyuan Zhou, Ying Li

**Affiliations:** a Acupuncture–Moxibustion and Tuina School, Chengdu University of Traditional Chinese Medicine; b Chengdu Pidu District Hospital of TCM/The 3rd Clinical Medical Hospital of Chengdu University of TCM; c Graduate School, Chengdu University of Traditional Chinese Medicine, Chengdu, China.

**Keywords:** acupuncture, dose-related, in vitro fertilization, meta-analysis, protocol

## Abstract

**Background:**

Previous studies have given an inaccurate assessment of the role of acupuncture in in vitro fertilization (IVF). We will use acupuncture doses as an entry point, discussing the dose-related effects of acupuncture therapy in women undergoing IVF.

**Methods:**

This study will search the following database: EMBASE, PubMed, Web of Science, the Cochrane Central Register of Controlled Trials (CENTRAL), and 4 Chinese databases. All databases will be searched from the date of database establishment to January 31, 2019. In addition, we will search possible studies which were included in previous meta-analyses. The primary outcomes are the clinical pregnancy rate (CPR) and the live birth rate (LBR). The secondary outcomes involved the biochemical pregnancy rate (BPR), the ongoing pregnancy rate (OPR), serum hormone level, the incidence of ovarian hyper-stimulation syndrome (OHSS), the cycle cancellation rates, and adverse events (AEs). After checking and integrating the raw data, we will use a 2-step to conduct the meta-analysis. Firstly, we will assess the effect of acupuncture on in vitro fertilization and embryo transfer (IVF-ET). Secondly, the meta-analysis will be performed for studies with similar total number of treatment sessions to investigate the dose-related effects of acupuncture. RevMan V.5.3 statistical software will be used for meta-analysis. If it is not appropriate for a meta-analysis, then a descriptive analysis will be conducted.

**Results:**

This study will investigate the relationship between pregnancy outcomes and the doses of acupuncture therapy in women undergoing IVF, and answer whether a higher-doses of acupuncture treatment will contribute to a better outcome of IVF-ET.

**Conclusion:**

The funding of this meta-analysis may provide convincing evidence for clinicians, benefitting more patients who crave children.

**INPLASY registration number:**

INPLASY202070072

## 1 Introduction

As a global public issue, infertility is explain as a disease of the reproductive system defined by the failure to achieve a clinical pregnancy after 12 months or more of regular unprotected sexual intercourse.^[1]^ Under the influence of environmental, biological, personal lifestyle, and social psychological factors, more and more families are suffering from infertilityfile://D:\Program Files (x86)\Youdao\Dict\8.8.1.0\resultui\html\index.html - /javascript:;.^[2]^ A World Health Organization's (WHO) report^[3]^ has shown that, in 2010, there were estimated 48.5 million couples were infertile around the world. In recent years, significant advances have been made in the treatment of infertility due to the advent of assisted reproductive technologies (ART). In vitro fertilization and embryo transfer (IVF-ET) is an important component of assisted reproductive technology. Over 300,000 ART cycles were performed in Europe during 2013. For all in vitro fertilization (IVF) cycles, the clinical pregnancy rate (CPR) per aspiration and per transfer were stable with 29.6% and 34.5%, respectively.^[4]^ However, due to lower endometrial receptivity, poorer embryo development, and a combination of other factors, CPR following IVF-ET remain low.^[5]^ Even some couples repeatedly failed in implantation.

Acupuncture, as a characteristic treatment of traditional medicine in China, is becoming more and more accepted worldwide because of its safety, effectiveness, and low side effects in clinical practice. Since 1990s, Stener-Victorin et al^[6]^ and Paulus et al^[7]^ reported that acupuncture could improves CPR in women undergoing IVF-ET. Studies^[8–11]^ have shown that acupuncture can significantly improve endometrial tolerance, regulate local blood circulation, and relieve anxiety, creating favorable conditions for embryo implantation. The application of acupuncture in the field of assisted reproduction has been receiving increasing international attention.

However, a confirmative and consistent result cannot be achieved by previous studies. Some studies^[12–14]^ showed that there was no significant difference in the pregnancy outcomes between acupuncture and sham acupuncture during IVF. Some systematic review^[15–17]^ have also been controversial about whether acupuncture can improve CPR and LBR in women who undergoing IVF. In summary, the impact of acupuncture on pregnancy outcomes remains uncertain.

We found that in studies with negative results,^[12–14,18]^ the number of acupuncture treatments was generally 1 to 3 times. However, women undergoing IVF have complex conditions and most of their primary illnesses require long-term acupuncture treatment. Some reviews^[19,20]^ suggested that during an ART cycle, the frequency, number of treatments, and timing of acupuncture were identified as important, at the same time, patients should start acupuncture treatment 3 to 6 months before IVF and receive >6 times treatments. Studies have shown that acupuncture treatment for 3 menstrual cycles can effectively improve the tolerance of the endometrium and create conditions for successful conception in women who have repeatedly failed to grow.^[10,21]^ We believe that the effectiveness of acupuncture is related to the courses as well as the frequency for the time of onset and accumulative effects. Therefore, we will investigate the relationship between pregnancy outcomes and the doses of acupuncture therapy in women undergoing IVF, and answer whether a higher-doses of acupuncture treatment will contribute to a better outcome of IVF-ET.

## 2 Methods and design

### 2.1 Study registration

This study protocol was registered in International Platform of Registered Systematic Review and Meta-Analysis Protocols (INPLASY) as INPLASY202070072. The protocol is structured in accordance with the Preferred Reporting Items for Systematic Reviews and Meta-analysis Protocols and Cochrane handbook.^[22,23]^

### 2.2 Search strategy

This study will systematically search EMBASE, PubMed, Web of Science, and the Cochrane Central Register of Controlled Trials (CENTRAL). In addition, we will also search 4 Chinese databases: China National Knowledge Infrastructure (CNKI), China Biomedical Literature Database (CBM), Wanfang Database, and China Science Journal Database (VIP database). All databases will be searched from the date of database establishment to January 31, 2019. Because of language limitation, we will only search and screen studies published in English or Chinese. Full articles or abstracts will be included. The following search terms will be combined for systematic search:

(1) “acupuncture”, “acupuncture therapy”, “electroacupuncture”, “auricular acupuncture”, “acupressure”, “transcutaneous electrostimulation”;
(2) “in vitro fertilization/IVF”, “intracytoplasmic sperm injection/ICSI”, “infertility”, “embryo transfer”, “assisted reproduction technology”;
(3) “randomized/randomised controlled trial”, “controlled clinical trial”.

The search strategy will be adjusted for different database requirements. Terms in Chinese will be used in Chinese databases. The search strategy of PubMed is shown in Table 1. Furthermore, we will search possible studies which were included in previous meta-analyses.

**Table 1:**
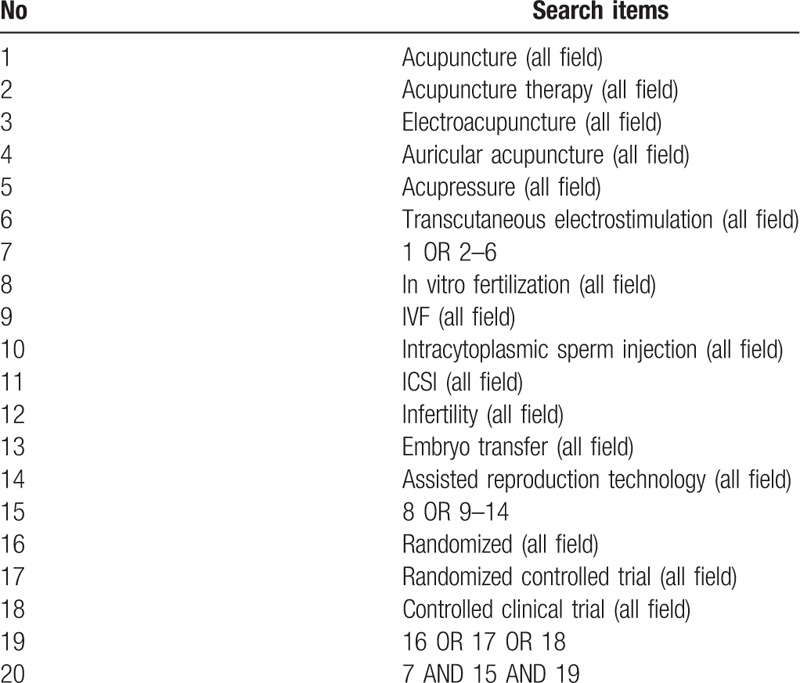
Searching items for identifying articles in PubMed.

### 2.3 Criteria for including studies

#### 2.3.1 Types of studies

To investigate the effectiveness of acupuncture in improving the IVF outcomes, we will include para-, randomized controlled trials (RCTs) that compared acupuncture therapy with sham acupuncture or placebo or no treatments (waiting list control). Studies assessing other effects of acupuncture in the period of IVF cycles, such as pain-relieving during the egg retrieval will not be included. To guarantee the quality, only the sample size was over 30 in each group will be included. We will exclude the following studies: retrospective studies, studies with crossover design, animal or in vivo experimental studies, case reports, or comments.

#### 2.3.2 Types of participants

We will include women undergoing IVF and age between 18 and 45. Patients underwent IVF with or without intracytoplasmic sperm injection (ICSI) will be included, no matter who were experiencing a fresh cycle of IVF, or having history of failure cycles before. Patients having other gynaecological diseases (i.e., polycystic ovary syndrome, diminished ovarian reserve, fallopian tube diseases, etc) will also be included.

#### 2.3.3 Types of intervention

The source of stimulation could be manual acupuncture, electroacupuncture, auricular acupuncture, warm needling, scalp acupuncture, intradermal needle, and transcutaneous electrostimulation (TENS). To control the heterogeneity, we will exclude studies reporting acupuncture therapy combined with Chinese medicine. Because the effectiveness of acupuncture therapy could not be evaluated.

#### 2.3.4 Types of comparisons

(1) Placebo controls: including needling in the control groups could either be with a sham needle device, such as Streitberger, Park placebo acupuncture devices, etc. Where skin penetration does not occur because the tip of the needle is blunted, normal acupuncture needles were applying at an area not recommended by TCM practitioners for fertility treatment, such as non-acupoints or non-specific points for fertility treatment, sham laser, placebo drugs, or other sham interventions.
(2) No acupuncture treatment, such as waiting list, which means that patients in the control group did not receive acupuncture treatment. Or patients received general care or usual care, which means that patients in the control group only receive advice and/or health education, such as exercise recommendations.

We will exclude the studies which applying Chinese medicine, or other methods that we can’t define the therapeutic effects as a control.

### 2.4 Types of outcome measures

#### 2.4.1 The primary outcomes

(1) Clinical pregnancy rate (CPR): defined by the presence of a fetal heartbeat at 6 to 7 weeks of pregnancy;
(2) Live birth rate (LBR): a baby born alive after 24 weeks gestation.

#### 2.4.2 Secondary outcomes

(1) Biochemical pregnancy rate (BPR): a positive human chorionic gonadotropin (hCG) serum or urine test 11 days after embryo transfer (ET);
(2) Ongoing pregnancy rate (OPR): pregnancy beyond 12 weeks of gestation which is confirmed by fetal heart activity on ultrasound;
(3) Endometria condition, such as receptibility, resistance index (RI) or pulsatility index (PI);
(4) Serum hormone level, such as follicle-stimulating hormone (FSH), luteinizing hormone (LH), FSH/LH ratio);
(5) The incidence of hyperstimulation ovarian syndrome (OHSS);
(6) Cycle cancellation rates;
(7) Adverse events (AEs).

### 2.5 Data collection and analysis

#### 2.5.1 Selection of studies

**Figure 1:**
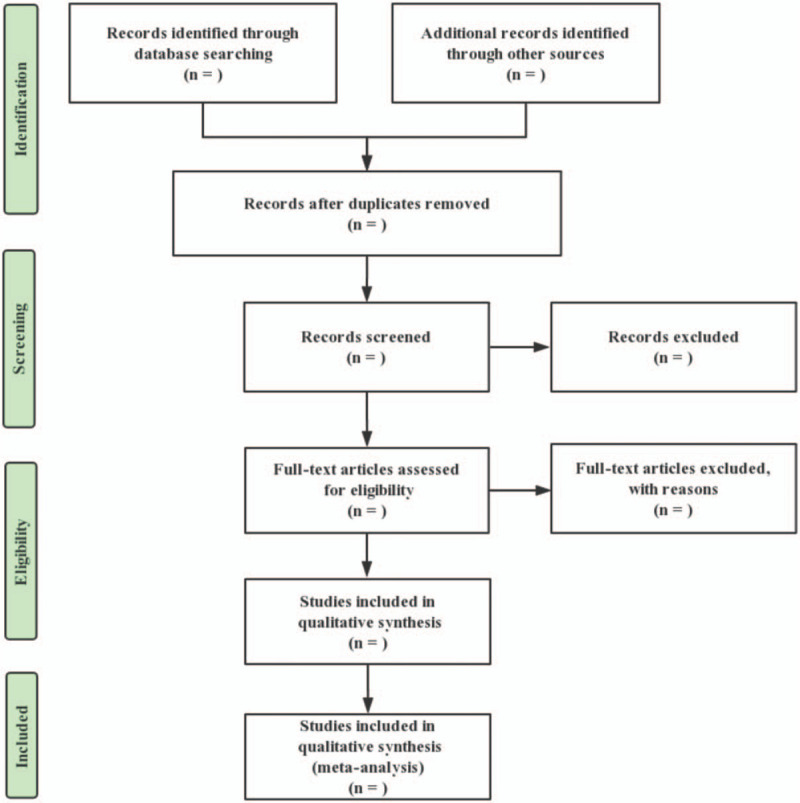
The PRISMA flow diagram of the study selection process. PRISMA = preferred reporting items for systematic reviews and meta-analysis protocols.

We will import the title, author, and abstract of the retrieved article from the database into Endnote V.X9 (Clarivate Analytics, Philadelphia, United States) and delete repetitive articles after checking by this software. Two reviewers (ZZH and WX) will independently go through article titles, abstracts, and keywords one by one to filter out articles that meet the inclusion criteria. Eligible full text will be further assessed while excluded studies will be recorded and interpreted. The results will be cross-checked by 2 reviewers. Any disagreements will be resolved by consensus. Further arguments will be arbitrated by a third reviewer (ZQH). The study flow chart is shown in Fig. 1.

#### 2.5.2 Data extraction and management

Two reviewers (YZQ and HTH) will independently check the eligibility of the included studies and extract data according to a predesigned data extraction form according to the Reporting Interventions in Clinical Trials of Acupuncture (STRICTA),^[24]^ which includes the following information: the general information of the studies, study characteristics, participants, interventions and controls, outcomes, results, adverse events, main conclusions, ethical approval, and other information. In order to investigate the doses related effects of acupuncture, we specifically document the number and frequency of acupuncture sessions, duration of treatment and the time window of acupuncture treatment. All the information from extraction form will be recorded in an Excel file. Reviewers will be trained before extraction and reach a common consensus to guarantee the consistency. Any discrepancy will be resolved through discussion between the 2 reviewers, or judged by a third reviewer (ZQH). If the reported data are insufficient, we will contact the author of the studies for consultation and solution.

#### 2.5.3 Assessment of risk of bias in included studies

Two reviewers (ZZH and WX) will independently evaluate the methodological quality of included studies by using the Cochrane Collaboration's tool for assessing risk of bias (Cochrane Manual V.5.1.0). The following domains will be accessed: random sequence generation, allocation sequence concealment, blinding, data integrity, selective reporting, and other sources of bias (such as study design, baseline similarity of groups, etc). The assessment results will be divided into 3 levels: low risk, high risk, and uncertain risk. In the process, the discrepancy will be discussed by the 2 reviewers to reach an agreement, or judged by a third reviewer (ZQH).

#### 2.5.4 Measures of treatment effect

For dichotomous data, such as CPR, LBR, BPR, OPR, and the rate of AEs, we expressed the results for each study as the risk ratios (RRs) with 95% confidence intervals (CIs). And for the continuous data, such as the number of retrieval eggs, blood hormone level, and so on, we expressed the results as the difference or standard mean difference (SWD) with 95% CI.

#### 2.5.5 Dealing with missing data

We will attempt to obtain missing or inadequate trial data by contacting the first author or correspondent author of the included study by email. If no additional data is available, we will analyze the existing data and discuss the potential impact of missing data.

#### 2.5.6 Assessment of heterogeneity

The heterogeneity will be evaluated by using both the *I*^2^ test and *P* value of the chi-square test of heterogeneity. When the *I*^2^ test is <50%, the study is not considered to have a large heterogeneity. When the *I*^2^ values are above 50%, there is significant heterogeneity between trials. To investigate the contributors to the heterogeneity, we will use sensitivity and subgroups analysis. If the heterogeneity is too high, the meta-analysis will not be performed, we will conduct a descriptive systematic review.

#### 2.5.7 Assessment of reporting biases

We will use the contour-enhanced funnel plot to assess the risk of publication bias within each pairwise comparison. If a sufficient number of studies are included (at least 10 trials), we will use funnel plots to assess reporting bias. If the funnel plot is asymmetric, Egger regression test will be used.^[25]^

#### 2.5.8 Data synthesis

In our review, the effect of acupuncture on IVF-ET will be assessed in first step. In order to investigate the dose-related effects of acupuncture, the meta-analysis will be performed for studies with similar total number of treatment sessions. Among the included studies, the total number of acupuncture treatment sessions will be divided as follow: low dose stimulation (1–3 treatments); medium dose stimulation (4–10 treatments); medium-high dose stimulation (11–30 treatments); high dose stimulation (>30 treatments). RevMan V.5.3 statistical software will be applied for data synthesis. Statistical analyses will be performed with RevMan V.5.3 statistical software to present direct and indirect comparisons between acupuncture treatment and controls. For dichotomous data, such as CPR, LBR, BPR, OPR, and the rate of AEs, we expressed the results for each study as the risk ratios (RRs) with 95% confidence interval (CIs). And for the continuous data, such as the number of retrieval eggs, blood hormone level, we expressed the results as the difference or standard mean difference (SWD) with 95% CI. According to the suggestions from the Cochrane Handbook, we will use a fixed-effects model for data synthesis if the *I*^2^ value is <50%; otherwise, we will use a random-effects model for data synthesis.

#### 2.5.9 Subgroup analysis

We plan to conduct the following subgroup analyses of the main results to explore the stability of the results across different study subgroups.

(1) Different time points of acupuncture treatment: acupuncture began 3 menstrual cycles before transfer; acupuncture began 3 menstrual cycles before transfer; acupuncture began from day 21 of the previous cycle until the day of human chorionic gonadotrophin (HCG) administration; acupuncture on the day of embryo transfer, etc;
(2) Different types of acupuncture treatment: such as manual acupuncture, electroacupuncture, TENS, etc;
(3) Different types of control: such as sham acupuncture or placebo;
(4) Different underlying diseases: such as PCOS, POF, etc

#### 2.5.10 Sensitivity analysis

To ensure the stability and reliability of the results, a sensitivity analysis will be performed. These will be based on different statistical approach, different heterogeneity quality, and different sample size. Excluding the studies which were poor quality or potential contributors to heterogeneity, the meta-analysis will be reused. We will compare the results and discuss.

#### 2.5.11 Grading the quality of evidence

The grading of recommendations assessment, development, and evaluation will be used to evaluate the quality of SRs. We will assess the risk of bias, inconsistency, indirectness, inaccuracy, and publication bias. The results of the evaluation were divided into 4 levels: high, moderate, low, or very low.

## 3 Discussion

The results of a single clinical study are often insufficient to draw definitive conclusions and remain inaccurate estimates of the true effect of the hypothesis. Meta-analysis is a great way to resolve uncertainty when report disagree and assist the applicability of the findings to a wider range of patients and treatment options.^[26]^

Most infertility patients tend to have underlying conditions. Several studies have shown that acupuncture is effective for conditions that cause infertility,^[27–29]^ and that all of these chronic conditions need multiple courses of acupuncture treatment.^[27,30]^ At the same time, menstruation, as a physiological phenomenon of the female cycle, is closely related to ovulation and has a natural cycle rhythm. Therefore, the acupuncture treatment of diseases related to the menstrual cycle should also be combined with the menstrual cycle.^[31]^ Previous studies have ignored the cumulative effects of acupuncture. Our study will address this question and attempt to explain whether acupuncture can achieve the goal of improving pregnancy outcomes through the treatment of underlying conditions. There are also some limitations in this systematic review. Firstly, due to language limitations, we can only include articles in Chinese and English; secondly, because some articles do not give the exact number of acupuncture treatments, we can only estimate the approximate range based on the descriptions in the text. Thirdly, we may have overlooked the discussion of the relationship between acupoints compatibility and the therapeutic role of meridian acupoints based on traditional Chinese medicine theory. Lastly, there is a high possibility that a large heterogeneity comes from the different acupuncture methods, treatment durations. Therefore, descriptive SR but not quantitative SR will be necessary.

With the development of complementary alternative medicine, acupuncture has been widely used in the field of assisted reproduction. We sincerely hope that the funding of this review could provide convincing evidence for clinicians, benefitting more patients who crave children.

## Author contributions

**Conceptualization:** Qianhua Zheng, Zihao Zou, Ying Li.

**Data curation:** Xin Wen, Zihao Zou, Zuoqin Yang, Tinghui Hou, Qianhua Zheng

**Formal analysis:** Qianhua Zheng, Siyuan Zhou.

**Investigation:** Qianhua Zheng, Ying Li, Xinyu Hao.

**Methodology:** Qianhua Zheng, Xinyu Hao.

**Project administration:** Qianhua Zheng, Ying Li.

**Supervision:** Ying Li.

**Writing – original draft:** Zihao Zou, Qianhua Zheng.

**Writing – review & editing:** Xinyu Hao, Ying Li
